# Opportunities, risks and challenges in global mental health and population neuroscience: a case of Sino-German cooperation

**DOI:** 10.1007/s00406-020-01176-1

**Published:** 2020-07-29

**Authors:** Shuyan Liu, Sabine Müller, Raymond J. Dolan, Xudong Zhao, Jialin C. Zheng, Andreas Heinz

**Affiliations:** 1 Department of Psychiatry and Psychotherapy, Charité – Universitätsmedizin Berlin (Campus Charité Mitte), Berlin, Germany; 2 Max Planck Centre for Computational Psychiatry and Ageing Research & Wellcome Centre for Human Neuroimaging, University College London, London, UK; 3 Pudong Mental Health Centre, Tongji University School of Medicine, Shanghai, China; 4 Center for Translational Neurodegeneration and Regenerative Therapy, Shanghai Tenth People’s Hospital affiliated to Tongji University School of Medicine, Shanghai, China; 5 Collaborative Innovation Center for Brain Science, Tongji University, Shanghai, China; 6 Department of Pharmacology and Experimental Neuroscience, University of Nebraska Medical Center, Omaha, USA; 7 Department of Pathology and Microbiology, University of Nebraska Medical Center, Omaha, USA

**Keywords:** Large-scale cohort studies, Ethics, Data security and privacy, Laws and regulations, Patient care, Population neuroscience

## Abstract

Large scale prospective cohorts have now been established across several countries, and continents, and among the aims include an assessment of the developmental trajectory of mental disorders. This level of international cooperation helps transfer research findings to new social contexts as well as enabling an assessment of which findings can be replicated, and which interventions are most effective, in different social and cultural settings. However, data sharing across different regional and national health care systems requires a careful consideration of different standards in ethical research, data protection and patient care, including respect for patients’ rights, in cooperating jurisdictions. In our review, we discuss ethical, legal and practical challenges associated with such cooperation with a focus on research participants, specifically patient recruitment, by considering the instance of China and Germany. Our broader aim is to promote international cooperation by identifying key challenges that arise in international cooperation, and to facilitate an exchange in relation to legal and practical approaches.

## Introduction

Large-scale, long-term, prospective cohort studies are invaluable in fostering a deeper understanding of the relative contributions and potential interactions of environmental factors and genetic predisposition, including epigenetic mechanisms underlying psychiatric disorders. These questions can be addressed powerfully through population neuroscience [30]. The IMAGEN study (https://imagen-europe.com, an often cited example [40], dates from 2007 and represents the first multisite genetic-neuroimaging study of individual variability in trait impulsivity, reinforcement sensitivity and emotional reactivity that aims to characterize comprehensibly the full spectrum of psychiatric disorders. Over more than a decade, the study has recruited over 2000 adolescents (14 years of age), with longitudinal follow-ups at ages 16, 19 and 22 involving acquisition of psychometric tests, neuroimaging and other biomarkers [34]. Neuroimaging data has been acquired at eight sites in the UK, Ireland, France and Germany [40]. Apart from the well-known Neuro Science in Psychiatry Network (NSPN) study from the University of Cambridge and University College London (https://www.nspn.org.uk, [23], the IMAGEN study is the only published large-scale cohort that includes both structural and functional magnetic resonance imaging dataset of subjects from the beginning of adolescence through into young adulthood [40]. A series of prospective cohort studies have subsequently been developed in other continents as part of the so-called Global Imaging Genetics Initiative in Adolescence (GIGA, involving collaborators in the United States, India, UK, Europe and China, that aim to ascertain impact of environment, ethnicity, culture and society on mental health [39]. In addition to standard metrics, GIGA assesses environmental factors using mobile-based technology with collection of real-time data whilst an analysis of satellite data, once set up, can be used to index features of urbanization over time and across geographical regions.

Systematic comparison of data across sites has the potential to identify biomarkers relevant to global mental health. Prospective cohort studies avail of ambulatory assessments for experience sampling as well as the mapping of environmental risk factors in everyday life [17]. This provides a unique opportunity to assess cultural and regional differences in social and environmental factors (e.g., stress exposure, failures in the caregiving environment, and substance use) pertaining to mental health [17]. Concerted international efforts are in train to streamline and harmonize these cohorts, at least with regards to studies on the clinical and psychometric characteristics of patients, as well as structural and functional neuroimaging techniques and procedures [39].

However, there are significant potential pitfalls arising out of such cross-border jurisdiction and cross-cultural enterprises although ethical standards are established by international conventions like the Declaration of Helsinki of the World Medical Association [3], the ethical guidelines of the Council for International Organisations of Medical Sciences [29], the Convention of Biomedicine of the European Council [7]. These pitfalls have not been clearly addressed in the literature, and they are the primary focus of this review.

First and foremost, there is a concern regarding data security and privacy, particularly with respect to ambulatory assessments, where these may include geolocation and information relating to behaviours such as substance and illicit drug use. Secondly, ethical requirements for research vary considerably across countries and this variability poses a challenge when conducting international studies. Finally, there is a concern that as soon as patients with mental health problems are involved in clinic research, patients within institutional care settings in in particular may feel undue pressure to participate in research, or harbour unfounded fears that findings emerging from study participation might negatively affect their status. We address these questions by considering cooperation with Chinese scientists regarding a planned establishment of a “Sino-German Mental Health Centre”.

## The pitfalls of international cooperation

### Ethics committees and review boards in Germany and China

Research ethics committees (Institutional Review Board, IRB) are required by all international ethical standards governing research involving human participants, as well as by local law in many jurisdictions. They are responsible for ensuring the protection of rights, safety and well-being of participants involved in clinical research. Unsurprisingly, considerable variation exists between ethics committees of different countries, which is a challenge for researchers wishing to conduct international studies, particularly when local ethical norms of one country are inconsistent with’ espoused universal ethical principles [25].

In Germany, all human subject research is required by law to acquire full ethical approval [12]. Ethics committee members in Germany come from a variety of academic backgrounds, which includes lay members and an appropriate gender balance. Thus, according to Article 41 of the German Medicinal Product Act (“Arzneimittelgesetz”, AMG), ethics committees should be constituted to include a balanced gender ratio (https://www.jusline.at/gesetz/amg/paragraf/41). According to the EU Clinical Trials Regulation from 2014, a lay member or patient representative has to be member of each research ethics committee. In Germany, committees are mandated to scrutinise the potential benefits for patients as well as the importance of the proposed research, weighing these considerations up against risks and burdens for participants. To perform an adequate risk/benefit assessment, committee members in Germany do not base their assessment solely on information provided in a protocol, but also actively seek out additional information, consult experts and exchange information with other committees. In the case of a large-scaled multisite cohort study in Germany, a local ethics committee may approve research after an affirmative recommendation has been received from a lead site, where the proposal was initially evaluated. For example, the multisite IMAGEN study was approved by a local research ethics committee at each national site in depth and then reviewed further by data protection officers at each site.

For China, it has been questioned whether scientists adhere to high ethical standards compared, for example, to those in Europe [55]. In fact, both the National Natural Science Foundation of China and Chinese Academy of Sciences have long established detailed regulations and guidelines [55]. Different types of institutions and authorities in China vary in the composition of their research ethics committee as to number, gender and professional background, and the statutory body [50]. Regarding the gender balance of ethics committee members, it was required in Chinese regulatory documents “Guiding Principles of Ethical Review of Drug Clinical Trials” and “Norms on the Quality Management for the Clinical Trials of Medical Devices” issued by the State Food and Drug Administration in 2010 and in 2016 respectively, while most regulatory documents issued by China’s relevant authorities did not mention it [50]. China's National Health and Family Planning Commission issued one of the most detailed provisions in “Measures for the Ethical Reviews of Biomedical Research Involving Humans (2016)” [50]. It stipulates that ethics committee members of medical and health institutions should be chosen from different fields including biomedicine, ethics, jurisprudence, sociology and the general public and should consist of no less than seven people, with lay members as optional [50]. In Shanghai, in 2008 52% of 33 public hospitals had engaged lay members for their ethics committees [53]. However, these rules are not always rigorously executed across the entire country. Many research institutions in China have not established objective and reliable mechanisms to deal with ethical issues and are often reluctant to deliver a definitive verdict on misconduct cases or impose appropriate punitive measures when there are violations [55]. This institutional tolerance and the system that evaluates a researcher mainly by the number of publications in both Germany and China, is a potential contributor to research misconduct [11, 33].

Ambulatory assessments with apps that provide geolocation as well as brain-inspired artificial intelligence is revolutionizing neuroscience, and this raises serious ethical concerns such that international consensus needs to be reached to take account of the unique characteristics of individual participating countries [15]. It is gratifying that both German and Chinese governments have provided explicit support for neuroethics research. An example is the German Federal Ministry of Education and Research (BMBF), together with European Research Area Networks fund joint transnational projects on Ethical, Legal, and Social Aspects (ELSA) of Neuroscience (https://www.neuron-eranet.eu). In China, there is an ongoing project [48] aiming at evaluating ethical issues related to the convergence of nanotechnology, biomedicine, information technology, and cognitive science (NBIC) [37] to ameliorate human capabilities [10, 47]. The ethical issues of this project are especially relevant for cognitive neuroscience research following the launch of the China Brain Project [32], which has clear priority objectives for integrating an ethical framework, addressing relevant ethical concerns, and planning for solutions [32].

As cultural factors can influence the extent and pace at which each society adapts to the international ethical guidelines, the International Brain Initiative (https://www.internationalbraininitiative.org) has been established to coordinate efforts across existing and emerging national and regional brain research initiatives. In this contexts, Global Neuroethics Summit Delegates have listed key ethical questions to guide ethical research [2].

### Data privacy, data security and compliance risk

Personal data privacy protection is a fundamental human right, and therefore it must be rigorously guaranteed by the research community. Many recent examples of unethical research practices have involved unauthorised collection and/or (mis)use of personal data, resulting in enforcement action by regulators [44]. In the European Union (EU), all research projects need to stay compliant with the General Data Protection Regulation 2016/679 (GDPR). Personal data must be processed in compliance with the EU and national data protection laws. Thus, research proposals that involve personal data must also comply with relevant ethical-legal principles. In light of the GDPR, some institutions in Germany have appointed a data protection officer to be involved in all stages of a project regarding data privacy issues. At these institutions (e.g., Charité–Universitätsmedizin Berlin), the ethics committees require separate project reports from this local data protection officer on all human studies in relation to a potential violation of data protection regulations. This extends to providing a complete list of internet service providers involved in handling data, users of such databases, adherence to pseudonymisation and anonymisation and time limits for erasure or review of stored data. However, surprisingly some institutions do not have a data protection officer. In this case, instead of specifically requiring external review, ethics committees at these institutions (e.g., Technical University of Dresden and Central Institute of Mental Health in Mannheim) have at least some member who has basic competence in data protection. With GDPR entering into force in 2018, the stage is set for international debate on Big Data sharing and international data transfers. Where complex, sensitive or large-scale data processing is envisaged or data are to be transferred outside the EU, consultation with the data protection officer is required regarding compatibility in data protection arrangements with respect to the host institution’s policies and relevant applicable legislation.

Furthermore, the right to the protection of personal data is enshrined in the Article eight under the Charter of Fundamental Rights of the EU, where each individual has the right to be informed about the collection and use of their personal data. For research, the EU has published a document on ethics and data protection in 2018 (https://ec.europa.eu/research/participants/data/ref/h2020/grants_manual/hi/ethics/h2020_hi_ethics-data-protection_en.pdf). It prescribes that in research settings, data protection imposes obligations on researchers to provide research subjects with detailed information about what will happen to personal data collected. It also requires the organisations processing the data to ensure that the data are properly protected, minimised, and destroyed when no longer needed.

China’s data privacy framework is splintered across rules enshrined in more than 200 laws, measures and sector-specific regulations. The Cyber Security Law, effective from 1 June 2017, provides for the first time a framework for comprehensive regulations of data protection in the form of national-level legislation. However, it does not spell out clearly the key requirements involving consent, anonymization, and securing personal information, and these questions were not addressed until 2018. In March 2018, China’s National Information Security Standardization Technical Committee (TC260) issued a recommended national standard (“GuoBiao/Tuijian”, GB/T), the Personal Information Security Specification GB/T 35,273–2017 (PIS Specification) [31]. Like the GDPR, China’s PIS Specification includes guidance on user consent, data protection, data access, the obligation of disclosure, and the evaluation of data security, but overall it is more permissive. For instance, Article six of the GDPR sets out six conditions (user consent, contract, legal obligation, vital interests, public task and legitimate interests) and at least one of these must apply in which personal data processing is legally permitted (https://eur-lex.europa.eu/legal-content/EN/TXT/?qid=1528874672298&uri=CELEX:02016R0679-20160504).

In China, the PIS Specification only lists four types of personal data (basic personal information, personal identity information, personal health and biometric information, personal education and work information) which data controllers are not allowed to process (https://www.datalaw.io/index.php/2019/08/05/the-right-of-access-of-access-to-personal-data-in-the-largest-global-internet-digital-markets/). Also, the PIS Specification is not mandatory, but only a recommended standard that cannot give Chinese citizens any rights to protect their privacy [31]. This is quite different from a Western notion of data privacy: China’s legislation on personal information protection may consider balancing business, government, society and science interests and calls for the importance of establishing the right to privacy. Nevertheless, China has made strides with regards to data protection, inspired by worldwide expertise and experience [48]. Recognizing the necessity to enhance patient safety and standardization of clinical trials regulation, the China Food and Drug Administration strengthened the encryption of patient information and emphasized that comprehensive informed consent is a prerequisite for clinical trials in its recently published amendment to laws and regulations on clinical trials of medicine and medical devices, in 2017 (https://english.nmpa.gov.cn/2019-12/16/c_432394.htm).

### Inadequate treatment and poor patient care

A key obstacle to ethically informed research on patients with mental disorders is undue pressure that is imposed on patients to consent to study participation, particularly within institutional settings. On the other hand, a current focus on patient-centred approaches to research and treatment allows patients to get involved in making their own decisions [19]. However, this approach is not always safeguarded in clinical practice and research. There are still only exceptional examples of efforts to engage patients in planning and discussing of mental health research [38]. In order to achieve more active patient participation in international research process, there is a need to improve the level of information that patients and society as a whole have on research objectives and processes. The goal here is to promote and measure the impact of patient participation in research and health care and to reduce undue pressure on patients to participate in studies.

In Germany, the National Association of Psychiatrists and Psychotherapists, DGPPN, has a so-called Trialogue Board that includes representatives of the major organizations of patients and relatives, who discuss national mental health care practices and policies together with professionals. There are also increasing opportunities for patients to participate more actively in the entire research process. For example, in a Collaborative Research Centre (TRR 265) in Germany (https://sfb-trr265.charite.de/en/), a stakeholder board was established that includes representatives of organisations of patients and relatives as well as public health care. In China, mental health policy implementation at local level do not capture the views or knowledge of all key stakeholders [54]. There is a great need to involve stakeholders including mental health professionals, parents, social services, religious leaders, and educators [54]

In spite of these efforts, a persisting lack of patient involvement in clinical research is the norm in many countries. This might, in part, be explicable by differences in the relative emphasis assigned to ethical principles (respect for autonomy, beneficence, non-maleficence, and justice) guiding the doctor-patient relationship [38, 48]. The traditional relational model, based on a principle of beneficence, which in turn is based on the authority of the doctor, may contribute to the fact that research is often performed for patients, but not with them. Therapeutic misconceptions, the absence of patients in many Institutional Review Boards (IRBs), poor quality of information provided to participants, and a low level of patient participation in establishing research priorities and study design are all elements highlighting how a beneficence principle predominates in respective national or local research fields [38]. In many countries and settings, doctor-patient relationships are changing, and concepts such as shared decision making, and patient empowerment are acquiring increasing importance [19]. Accordingly, the doctor-patient relationships should be more strongly grounded on the principle of autonomy. Autonomous decisions are not possible as long as patients lack the necessary information. Providing the general public with more information about the aims of research can help to promote autonomy, generate trust and promote a wider participation in research. Informed consent is a key aspect in any research setting that assures that the patients’ autonomy is respected.

However, the situation can be complex in mental health institutions. Under national law in China, as well as in Germany, patients with certain special conditions can be admitted involuntarily, or against their will, to a psychiatric hospital. Researchers in psychiatry have to evaluate carefully for each patient, who has been admitted involuntarily to a psychiatric hospital, whether he or she can give informed consent to participate in research whilst being involuntarily detained [46]. No patient should be coerced to participate in research, even if a surrogate (legal guardian) agrees on their behalf [35]. But even without overt coercions, patients who are unable to give informed consent should not be used as research subjects as a general rule.

In Germany, there are two legal regimes – the guardianship law (which is a national law) and the mental health laws that apply in each of the 16 German federal states [56]. These laws regulate the mental health care system for people with mental disorders, as well as involuntary admission to psychiatric hospitals and compulsory treatments. Different laws in each of the German federal states (Bundesländer) regulate admission and compulsory treatment for acute cases [14]. The German guardianship law (Civil Code) covers non-acute care especially for those who are unable to care for themselves [56]. In China, a national Mental Health Law was enacted in 2012 [5]. This law guarantees, for the first time in China, the right to adequate treatment and provides legal protection in cases of malpractice [51]. The law requires informed consent from all mentally ill patients before they receive inpatient treatment and prohibits involuntary admissions unless those severely ill patients are deemed to have the potential to harm themselves or others [5]. Nevertheless, clinical practice differs widely [24]. The process of drafting, adopting and implementing such legislation provided an opportunity for raising public awareness and educating policy makers and society in general [51].

As local applications and implementations vary in both Germany and China, checking national regulations is not enough to safeguard patients’ rights. Therefore, we now discuss some differences in the clinical practice which are relevant for research in the following sections. A hidden pressure on patients to consent to participate in research can in particular be seen in patients who face long-term involuntary admission or feel the informal pressure to stay in hospital [49]. The duration of treatment varies substantially between countries [9, 41]. In Germany, most psychiatric hospitals offer support for a rather short period of time of usually up to, at most, several months, with the average length of stay being 23.8 days in 2017, according to Federal Statistical Office in 2018 (Data retrieved from the document on page 26, https://www.destatis.de/DE/Themen/Gesellschaft-Umwelt/Gesundheit/Krankenhaeuser/Publikationen/Downloads-Krankenhaeuser/grunddaten-krankenhaeuser-2120611177004.pdf;jsessionid=278997CAFAE3BE0C49EC28BC1C3D88E6.internet741?__blob=publicationFile). In addition, psychiatric hospitals often provide an integrated outpatient clinic, which facilitates early discharge from inpatient treatment. Furthermore, Germany offers partial inpatient treatment (*Tagesklinik*) to ease a transition from clinic to home. In these settings, patients spend the entire day at the clinic, but go about their everyday lives afterward and sleep at home.

In China, patients usually have a considerably longer stay in psychiatric hospitals. Statistics from 2013 to 2016 showed that inpatients in China spent an average of 240.2 days in specialized hospitals and 95.5 days in community health centres [41]. Many patients have to stay in the closed ward of a psychiatric hospital for a very long time because they either do not dare to leave or because their families are not willing to take them home [52]. Thus, there is urgent need to improve the spirit of humanistic care in mental health services [52], which could also improve the conditions for giving informed consent to study participation.

Furthermore, mental health services in China often suffer from severe understaffing and a scarcity of mental health facilities. Compared to the large population of China, the number of psychiatrists and nurses is exceedingly small. As of 2018, China’s National Health Commission reported there are only 40,435 registered psychiatrists and 101,282 registered psychiatric nurses; that is 2.9 psychiatrists and 7.3 psychiatric nurses per 100,000 population (Data retrieved from the website https://www.xinhuanet.com/health/2019-11/25/c_1125270551.htm). These numbers are much smaller than those in high-income countries with 12.7 psychiatrists and 23.5 psychiatric nurses per 100,000 population according to the Mental Health ATLAS 2017—World Health Organisation (Data retrieved from Fig. 3.2.4 and 3.2.5; https://apps.who.int/iris/bitstream/handle/10665/272735/9789241514019-eng.pdf?ua=1). Meanwhile, there are few social workers and psychotherapists in medical institutions, and the clinicians have nearly no time to communicate with patients and their families [52]. Moreover, psychiatric hospitals are generally poorly equipped and overcrowded compared to other medical facilities in China. Nearly 40 years ago, Allodi and Dukszta [1] reported that the Shanghai Psychiatric Hospital had 1,006 inpatients beds and 14 wards with an average of approximately 72 patients per ward. In 2019, a psychiatric hospital in Pudong New Area had an average of approximately 120 patients per ward. One important factor contributing to this insufficiency might be the low salary of mental health clinicians [52]. The per capita incomes of psychiatrists and psychiatric nurses are lower than those of their colleagues in other specialties at general hospitals, such as physicians and surgeons [52]. In China, there is an urgent need to expand the number of psychiatrists and related practitioners, and ensure quality in remuneration with respect to other specialties [52].

Moreover, ward settings can contribute to patient pressure on participating in a clinical study. In many countries, locked entrance doors in psychiatric wards are encountered even by voluntarily admitted patients [13, 36], and can be experienced as forced confinement, an unwanted dependence on staff and lead to emotional distress [16]. In China, most inpatient wards are permanently locked, even though many patients are not legally obliged to stay in the hospital [51]. Recently, some specialist wards in China have changed from completely closed type to more open wards, thus to better meet needs of the patients [42].

In Germany, legally admitted patients are traditionally admitted to locked wards [22], which regularly takes care of patients who voluntarily enter the hospital [6, 20]. However, in recent years, several clinicians and bodies advocate greater efforts towards implementing open door policies in psychiatry, which means treating legally committed patients on open, rather than locked, wards [4, 8]. While concerns have been voiced regarding a potential decrease in security and control [43, 45], several studies suggest that the risk of absconding or violent interactions is not increased in such settings [6, 20, 21]. Future studies should assess whether wards settings (open versus closed) or duration of hospital treatment affect patients’ autonomy when deciding whether to participate in a study offered by the same institution in which they are treated as inpatients.

## Conclusion

A population neuroscience perspective emphasizes a need to understand the evolution of human behaviour and the expression of mental well-being across multiple levels of research. There is now an increasing reliance on the use of large cohorts, including ambulatory assessments that monitor many daily life behaviours. These instruments are capable of generating powerful data of scientific value. However, specific types of data sets, such as those that use geolocation data, may intrude upon individuals’ privacy and influence their social interactions. Therefore, safeguards are needed to maintain participants’ autonomy and safeguard their privacy. Research cooperation across jurisdictions should start by reflecting on potential legal and ethical implications of international cooperation. We emphasize the need to ensure the continuing competence of international cooperation through setting an equivalence of baseline standards in legislation relevant to ethics of patient care across countries. We highlight that ethics and data protection measures vary considerably between Germany and China, and we surmise may do so across other continents. Furthermore, ethical frameworks that impinge on research practice differ considerably even across institutions within a jurisdiction, with studies indicating rules are not rigorously executed with equanimity across China [55]. With the advent of GDPR, the EU has established a uniform framework for data protection legislation, and it is notable that there are no equivalent data privacy laws in China. Likewise, ward settings and durations of voluntary and involuntary treatment differ widely between Germany and China [6, 18, 26–28, 48, 51], and there is a striking lack of empirical research in both jurisdictions that assesses the differential impact of inpatient treatment on patients’ autonomy, and their participation in research.

The Department of Psychiatry and Psychotherapy at the Charité Campus Mitte plans to establish a “Sino-German Mental Health Centre” together with Tongji University School of Medicine in Shanghai. The proposed centre will address an urgent need for independent replication in large international cohorts across cultural settings, including promotion of patients’ rights in the context of research participation and engagement. This will focus on key issues including the role of ethics committees, data security regulations, patients’ rights, patient treatment settings including open or closed wards, public involvement in research design and grant development. Ultimately, the aspiration is to promote a unique perspective and experience of open science and data sharing sets a standard for a global health framework.
